# The Hospitals and Charitable Institutions of the United Provinces of India

**Published:** 1904-09-17

**Authors:** 


### THE HOSPITALS AND CHARITABLE INSTITUTIONS OF THE UNITED PROVINCES OF INDIA.

The total number of dipensaries in the united provinces of India at the end of 1902 was recorded as 495, being a gain of ten during the twelve months. A separate police hospital has been opened at Naini Tal, thus enabling the authorities to discontinue the objectionable practice of placing the police sick in the local charitable dispensary. Although it is very desirable that a new dispensary should be opened wherever there is an urgent need for it, some of these institutions have been started in late years on insufficient foundation. The minimum amount upon which a dispensary is allowed to be set up is the sum of 300 rupees promised locally. But district officers who have recommended the opening of new dispensaries with no more secure capital than this have experienced a lack of funds to equip and start the dispensary in an efficient condition. Therefore it has been impressed on district officers that the needful 300 rupees should be contributed by one or two persons only, so as to leave the subscriptions of the rest of the inhabitants free for the extra cost of maintaining their dispensary. In one case, where a dispensary was opened not long since, the needful minimum was guaranteed by 630 subscribers, none of whom had promised more than 8 annas as their annual contribution! It has been proposed that where the inhabitants of a village can by combining maintain a dispensary without Government aid, it would be better to support a private medical practitioner, instead of establishing a dispensary where the medical officer in charge has to attend gratis the whole subscribing population, a practice tending to pauperisation. But against their proposal is the fact that qualified native doctors of the hospital assistant class would not have a sufficiently large or lucrative clientèle to afford to buy the appliances now provided in dispensaries. Therefore, at present the dispensary is the only means of bringing the benefits of Western surgery and medicine to the people. The Inspector-General has been struck during his visits to the out-patients' departments of the various institutions of the United Provinces with two serious defects in the arrangements. First, the utter want of privacy in the treatment and examination of out-patients. In hardly a single case was there a couch handy and sheltered from the public gaze where either men or women could be examined. The benches or forms on which the patients sat were used for the purpose, and the Inspector-General was informed that the patients did not mind, and, in fact, liked such a state of affairs! In spite of this assertion, alterations are being made wherever possible, and it is hoped that when it is realised by the natives that privacy can be assured, a greater number of female diseases may be treated in the out-patients' departments.

Another grievance is the prevalent custom of giving only one day's supply of medicine at a time, so as to ensure the patients coming again the following morning, and thus increasing the number of attendances to be registered. This is, of course, a distinct hardship, more especially to weak and sickly patients who reside some distance from the dispensary, and has had the result of causing many to discontinue their attendance before they are really cured. Owing to the representation of the drawbacks of this system, the Lieutenant-Governor of the United Provinces has taken steps to impress upon the doctors at dispensaries that the Government attach no weight to statistical comparisons of attendance at the various dispensaries, and that in future treatment for two or three days is always to be given to each patient.

A considerable number of improvements to existing hospitals have been carried out during the year. At Lucknow, a fine European ward has been added to the Balrampur Hospital, well furnished and equipped, consisting of several private rooms, which seems to meet a want, as it has been almost constantly full since it was opened. The patients pay three to five rupees daily. An excellent operating room has also been arranged in the native part of this hospital, and the private wards for natives have been improved. The hospital was enriched during the year by a donation of 10,000 rupees from the Maharajah of Balrampur. At Agra, the fine new Lady Lyall Hospital, by providing fresh accommodation for female patients, has allowed the present building to be used for an ophthalmic hospital. Consequently, the Agra Medical School, with its attached hospitals and hostels for male and female students, is as complete and fine an institution as any that exists in the country. The Lieutenant-Governor gave a large sum of money during the year to supply the deficiencies in the surgical equipment of all district hospitals. This money was much needed, for at some stations the instruments were old and obsolete, dating from the early days of the last century. Others, though very expensive, were never used. With regard to the operation-rooms, many have had to be remodelled, as no good provision had been made for the main essential—proper light. Notably at Jhánsi, the pretty operating-room had narrow pointed windows and diamond glass panes, and resembled a nice little "oratory," with a correspondingly dim religious light! The visits of the lady doctors and female hospital assistants paid during the year to women at their homes in the bazaars were 3,833 at 37 centres.

